# The Fifteenth International Congress of Medicine

**Published:** 1906-05-05

**Authors:** 


### HOSPITAL ADMINISTRATION.

### CONSTRUCTION AND ECONOMICS.

### THE FIFTEENTH INTERNATIONAL CONGRESS OF MEDICINE.

The Lisbon Congress, like its fourteen predecessors, is now a thing of the past, and we can gather some lessons from it. The Portuguese, under the supervision of Professor Miguel Bombarda, the general secretary, have shown themselves to be very able organisers. The crush and confusion which attended the International Congress at Madrid were avoided by the simple expedient of allotting a separate bureau to each group of nationalities and placing these offices under the control of a lady resident in Portugal, but native of the country of those with whom she was called upon to deal. By this arrangement the English received their badges and communications from an Englishwoman, and the French from a Frenchwoman. The friction and loss of temper incident to a single bureau with the ordinary clerks imperfectly acquainted with several languages was thus avoided, and the courtesy of the ladies was sufficient to smooth all difficulties. The New School of Medicine at Lisbon has just been built, and was really inaugurated by the Congress. Its numerous and admirably proportioned rooms were excellently adapted for the purposes of the sectional meetings, but in too many cases the meetings were spoilt by the unpunctuality of the officers who, having announced the opening of a section for half-past eight, did not themselves attend for an hour or more afterwards. The sectional meetings were thus attended meagrely, for many who came early found the rooms empty, and went away to visit the hospitals and other institutions in the town. The visits to the hospitals and the arrangements at the medical school show that the medical profession in Portugal is advancing steadily, and has already made considerable progress along the lines followed by the most advanced nations. The wards in the hospitals are very large, bright, and well ventilated, and the patients seem comfortable. The nursing still needs improvement, though it has passed out of the hands of the religious sisterhoods.

The picnic or social side of the Congress presented greater attractions for the majority of the members of Congress than the scientific questions offered for discussion. Their Majesties the King and Queen of Portugal took an active interest in the proceedings of the Congress. They not only attended the opening meeting, but they invited a large party to dine at the palace. They attended the bull-baiting at Villa Francha—some 12 miles away from Lisbon—and they gave a garden party in the grounds of their private palace, which they attended personally. The bull-ring and the quarter-staff play at Villa Francha were distinctly interesting to Englishmen, as they enabled the visitors to see two forms of sport which delighted our forefathers; whilst to those who were more peacefully inclined the hospitality of Sir Francis and Lady Cook appealed more directly. Their house and grounds at Montserrat were thrown open to everyone, and a rare collection of the most valuable articles was seen in its setting of some of the most beautiful gardens in the world.

On Monday evening the Geographical Society gave an evening party, which was largely attended by members of the Congress. The morning of Tuesday was devoted to work in the sections and to the various hospitals, amongst others to the Leper Hospital of St. Lazarus. The existence of a special leper hospital in Lisbon is interesting in view of Mr. Hutchinson's contention that leprosy is caused by the consumption of badly preserved dry fish, for if the evidence of the shops is to be trusted, the poorer inhabitants of Lisbon must eat large quantities of dried ling, cod, and cuttle fish. The afternoon of Tuesday was devoted to the King's Garden Party in the grounds of his private palace at Necessidades. The guests were very numerous, and were received by their Majesties the King and Queen of Portugal, the Queen Dowager, and other members of the Royal Family, who attended in a stately procession, and made a very complete promenade amongst the guests. In the evening there was a Government reception, from which the majority of the British party were unavoidably absent, as the *Ophir* left Lisbon at seven o'clock in the evening. The two prizes awarded by the Congress triennially at each of its international meetings, called respectively the Moscow prize and the Paris prize, were gained by Professor Laveran and Professor Ehrlich for the excellence of their scientific work.

The work of the sections was continued on Wednesday, and the various hospitals and public institutions at Lisbon were inspected by numerous members of the Congress. The town of Lisbon gave an official reception in the evening, and on Thursday the Congress was closed in the usual formal manner.

The *Ophir*, with the majority of the British members of Congress on board, stopped a day at Oporto, and were most hospitably entertained by the British colony resident in the city. A reception committee came on board as soon as the ship anchored, and invited the whole of the members of the Congress to lunch about five miles up the river. The party, to the number of 150, were conveyed to the luncheon place by trams and boats, and were afterwards shown the "wine lodges," or places where port wine is matured before it is shipped away for home consumption. Twenty members of the Congress were afterwards entertained most sumptuously by the British Association of Oporto, and the party broke up at a late hour and with mutual satisfaction. A two-days' run through the Bay of Biscay and up the Channel brought the *Ophir* to an anchorage at Tilbury after a successful trip, which the greater number of the party seemed to have enjoyed.

